# Development of a Chinese version of the Western Ontario Meniscal Evaluation Tool: cross-cultural adaptation and psychometric evaluation

**DOI:** 10.1186/s13018-016-0424-8

**Published:** 2016-08-15

**Authors:** W. W. Tong, W. Wang, W. D. Xu

**Affiliations:** 1 Department of Orthopedics, Changhai Hospital, Second Military Medical University, 168 Changhai Road, Shanghai, 200433 China; 2 Department of Orthopedics, Chengdu Military General Hospital, Chengdu City, People’s Republic of China

**Keywords:** Meniscal pathology, Reliability, Validity, Cross-cultural adaptation

## Abstract

**Background:**

The Western Ontario Meniscal Evaluation Tool (WOMET) is a questionnaire designed to evaluate the health-related quality of life (HRQOL) of patients with meniscal pathology. Our study aims to culturally adapt and validate the WOMET into a Chinese version.

**Methods:**

We translated the WOMET into Chinese. Then, a total of 121 patients with meniscal pathology were invited to participate in this study. To assess the test-retest reliability, the Chinese version WOMET was completed twice at 7-day intervals by the participants. The construct validity was assessed using Pearson’s correlation coefficient or Spearman’s correlation to test for correlations among the Chinese version WOMET and the eight domains of Short Form-36 (SF-36), the Western Ontario and McMaster Universities Osteoarthritis Index (WOMAC), and the International Knee Documentation Committee (IKDC) score. Responsiveness was tested by comparison of the preoperative and postoperative scores of the Chinese version WOMET.

**Results:**

The test-retest reliability of the overall scale and different domains were all found to be excellent. The Cronbach’s *α* was 0.90. The Chinese version WOMET correlated well with other questionnaires which suggested good construct validity. We observed no ceiling and floor effects of the Chinese version WOMET. We also found good responsiveness for the effect size, and the standardized response mean values were 0.86 and 1.11.

**Conclusions:**

The Chinese version of the WOMET appears to be reliable and valid in evaluating patients with meniscal pathology.

## Background

The meniscus has been considered as a vital structure with various functions, such as transmission of the load, absorption of the shock, and stabilization and proprioception of the knee joint. The injury of the meniscus is a common source of pain, functional impairment, and even long-term articular cartilage deterioration of the knee [1]. Meniscus injuries are one of the most common injuries to the knee encountered by orthopedic surgeons today with an incidence of about 12–14 % [2]. It can be treated conservatively or surgically; surgeries that successfully repair or replace the meniscus are therefore likely to prevent or delay osteoarthritis progression [3, 4]. Due to its high morbidity and profound impact on health-related quality of life, meniscus injuries are a serious health and economic problem [5]. Health-related quality of life (HRQOL) questionnaires are used to evaluate patients’ general condition and offer a way to measure the effects of various therapies [6]. The meniscus injury HRQOL questionnaires enable physicians to take the patients’ perception into account to make a better therapy decision and assess the benefit of conservative and surgical interventions for patients with meniscal pathology [7, 8].

There are 11 commonly used knee-specific quality of life instruments: 5 for all kinds of knee disorders, 4 specific to anterior cruciate ligament ruptures, 1 specific to meniscal tears, and 1 specific to osteoarthritis of the knee [9]. The Western Ontario Meniscal Evaluation Tool (WOMET) is a disease-specific HRQOL questionnaire to measure physical symptoms, sports/reaction/work/lifestyle, and emotions of patients with meniscal pathology [9, 10]. There are 16 items representing three domains, and the sum score ranges from 0 (best) to 1600 (worst). The WOMET has been proven to be valid in patients with a degenerative meniscal tear and is widely used in several clinical trials [11–14]. It also has been translated into other languages like Turkish [15]. China has a population of nearly 1.3 billion, and Chinese is one of the most general languages in the world; however, we have not got a Chinese version of the WOMET so far.

Therefore, the purpose of this study was to perform a cross-cultural adaptation of the WOMET for Chinese people and to assess the psychometric properties of the translated version.

## Methods

### Translation and cross-cultural adaptation

The translation and cross-cultural adaptation were performed according to previously published guidelines [16, 17]. First, forward translation from English to Chinese was performed by three independent people who were native Chinese, two of them were orthopedic residents and the last person was a professional translator with no medical background. A consensus version was obtained after discussion of the three translators. The questionnaire was re-translated into English by a native-speaking English person blinded for the original English version of the WOMET. Then, we held an expert committee composed of all translators to resolve discrepancies. A final Chinese version was generated after pre-testing the pre-final version on 15 patients.

### Participants and statistical analysis

A total of 121 consecutive patients with meniscal pathology (52 men, 69 women) who underwent arthroscopic surgery for meniscal repair or resection were recruited from our hospital between October 2013 and December 2014. The population was in accordance with the criteria proposed by Terwee et al. [18]. Table 1 illustrates the clinical characteristics of these patients. The inclusion criteria were as follows: age >18 years, able to read and speak Chinese, patients with meniscus injuries diagnosed by magnetic resonance imaging (MRI), and two experienced knee surgeons. The exclusion criteria were as follows: patients with ligament injuries, such as anterior and posterior cruciate ligament; patients with history of leg surgery, infection, tumors, rheumatologic disease, or neurological or musculoskeletal disorders; patients who were unable or unwilling to complete the questionnaire. All the patients signed informed consent to participate in this study, and the clinical research was approved by the Local Ethics Committee of Changhai Hospital, SMMU (Shanghai, People’s Republic of China), and the reference number of the ethics committee is CHEC2013-194.

**Table 1:** Characteristics of participants

Characteristics	Total sample (*N* = 121)	Male (*n*1 = 52)	Female (*n*2 = 69)	*P* value^a^
Age (years; mean ± SD)	41.2 ± 14.3	41.0 ± 14.9	43.8 ± 15.4	0.337
Range	16–78	16–72	18–78	
Age groups, number (%)				0.844
≦20	15 (12.4)	8 (15.5)	7 (10.2)	
20–40	36 (29.8)	15 (28.8)	21 (30.4)	
40–60	44 (36.4)	19 (36.5)	25 (36.2)	
≧60	26 (21.5)	10 (19.2)	16 (23.2)	
Affected side, number (%)				0.855
Right	65 (53.7)	28 (53.8)	36 (52.2)	
Left	56 (46.3)	24 (46.2)	33 (47.8)	
BMI (kg/m^2^; mean ± SD)	25.6 ± 4.1	24.7 ± 4.1	26.1 ± 4.3	0.081
Symptom duration (months; mean ± SD)	15.6 ± 6.0	14.8 ± 5.4	15.9 ± 6.4	0.343
Range	1–36	1–33	1–36	

*BMI* body mass index

^a^Calculated by Student’s *t* tests for continuous variables and chi^2^ tests for categorical variables between males and females

Statistical analysis was performed using the Statistical Package for the Social Sciences (version 20.0, SPSS, Chicago, IL). All reported *P* values are two-tailed, and *P* values <0.05 were considered significant.

### Other instruments

To determine construct validity, the patients also completed the eight domains of Short Form-36 (SF-36), the Western Ontario and McMaster Universities Osteoarthritis Index (WOMAC), and the International Knee Documentation Committee (IKDC) score.

The SF-36 was used to measure general health status, and it contains eight domains: physical functioning, role-physical, bodily pain, general health, vitality, social functioning, role-emotional, and mental health. The SF-36 has been translated and validated in Chinese populations, we used the Chinese version, and the total scores vary from 0 (worst health status) to 100 (best health status) [19].

The WOMAC is a self-reported questionnaire specifically designed to evaluate the functional state of the knee or hip, and it contains three domains: pain (five items), stiffness (two items), and function (17 items). The data are standardized, generating scores for each dimension with a range from zero (best health status) to 100 (worst) [20]. The WOMAC has been translated and validated in Chinese population [21].

The IKDC score is a knee-specific rather than disease-specific outcome instrument designed for patients with a variety of knee conditions such as ligament injuries and meniscus injuries. It consisted of three domains: symptoms, function, and sports activity, and the total scores vary from 18 (worst) to 100 (best) [22, 23]. The IKDC had showed acceptable psychometric performance for outcome measures of meniscus injuries [24].

The participants were asked to complete the WOMET, the SF-36, the WOMAC, and the IKDC score when they first came to our outpatient room. Seven days later, they were asked to complete the questionnaires for the second time to determine the test-retest reliability when they came to our department to have the arthroscopic surgery. No medical intervention was provided during the period to minimize the clinical change. Six months after surgery, the participants were required to complete the WOMET for the third time.

### Acceptability, score distribution, and ceiling and floor effects

To evaluate acceptability, the patients were asked if there were any difficulties that had been encountered. The data were checked for missing or multiple responses. The presence of ceiling and floor effects was evaluated by calculating the percentages of the patients having the maximum or minimum score. There are no floor or ceiling effects if less than 15 % of the patients are having a minimum or maximum score based on the quality criteria and definitions [18].

### Reliability

Reliability test included evaluations for test-retest reliability and internal consistency. We calculated test-retest reliability by comparing scores of the first and second time, an intra-class correlation coefficient (ICC) was calculated to quantify test-retest reliability, and ICC >0.80 indicated excellent reliability [25]. Cronbach’s *α* was used to evaluate internal consistency, and when >0.7, >0.8, and > 0.9, the questionnaire is regarded to have acceptable, good, and excellent internal consistency, respectively [26]. Bland-Altman plots were used to describe the mean scores of the two assessments and differences between them. Each point indicates the difference in score of Chinese version WOMET for each patient between the two assessments (test and retest). The dashed line shows the 95 % (1.96 SD) limits of agreement. Analyze the distribution of the points and their relationship with the limits of agreement. More than 95 % of the points within the scope of limits of agreement were acceptable. Then, compared with the acceptable professional limits, if the limits of agreement are within the acceptable professional limits, it indicates good consistency between the two assessments (test and retest). Systematic bias can also be assessed according to the plots [27].

### Validity

By the Kolmogorov-Smirnov test, we found that the overall scale scores and all the subscale scores of the WOMET, IKDC scores, age, and BMI were all normally distributed. So we calculated the correlation with Pearson’s correlation. Subscale scores of SF-36 and WOMAC were abnormal distributed, so we calculated the correlation with Spearman’s correlation.

The construct validity was assessed using Pearson’s correlation coefficient (*r*) to test for correlations among the WOMET, WOMAC, IKDC score, and the SF-36. The *r* value >0.8 indicated excellent construct validity, and the correlations were judged as poor (*r* = 0–0.20), fair (*r* = 0.21–0.40), moderate (*r* = 0.41–0.60), and good (*r* = 0.61–0.80) in different values [10, 28]. We hypothesized that the physical symptoms and sports/reaction/work/lifestyle domains of the WOMET would correlate better with IKDC and several similar domains of the SF-36, and the emotion domain of the WOMET would correlate better with mental health domain of the SF-36.

### Responsiveness

To evaluate the responsiveness, we compare the preoperative scores and 6-month postoperative scores. Standardized response mean (SRM) was calculated by using the SD of the changes between preoperative scores and postoperative scores divided by mean of the changes. The effect size (ES) was calculated by using SD of the preoperative WOMET scores divided by the mean change between pre- and postoperative [18, 29].

## Results

### Translation process, acceptability, score distribution, and ceiling and floor effects

When the pre-final version was pre-tested on 15 patients, the patients were confused with the differences between “giving away” and “weakness.” After the discussion of all the translators, an easy-understanding Chinese version WOMET was generated. The patients did not have any difficulties in completing the Chinese version WOMET, and there were no missing or multiple responses. A total of 121 patients completed all the four questionnaires in the first and second assessments. The scores of the WOMET ranged from 240 to 1179, and no ceiling and floor effects were shown in Table 2.

**Table 2:** Score distribution and floor-ceiling effects of the Chinese version WOMET

Scale	Mean ± SD	Observed range	Theoretical range	Floor effect (%)^a^	Ceiling effect (%)^a^
Overall scale	716 ± 201	240–1179	0–1600	0	0
Physical symptoms	454 ± 133	0–820	0–900	0.8	0
Sports/reaction/work/lifestyle	117 ± 49	0–240	0–400	1.7	0
Emotions	145 ± 48	31–300	0–300	0	3.4

*WOMET* Western Ontario Meniscal Evaluation Tool

^a^Percentage of patients with the worst (floor effect) and the best (ceiling effect) condition

### Reliability

**Fig. 1:**
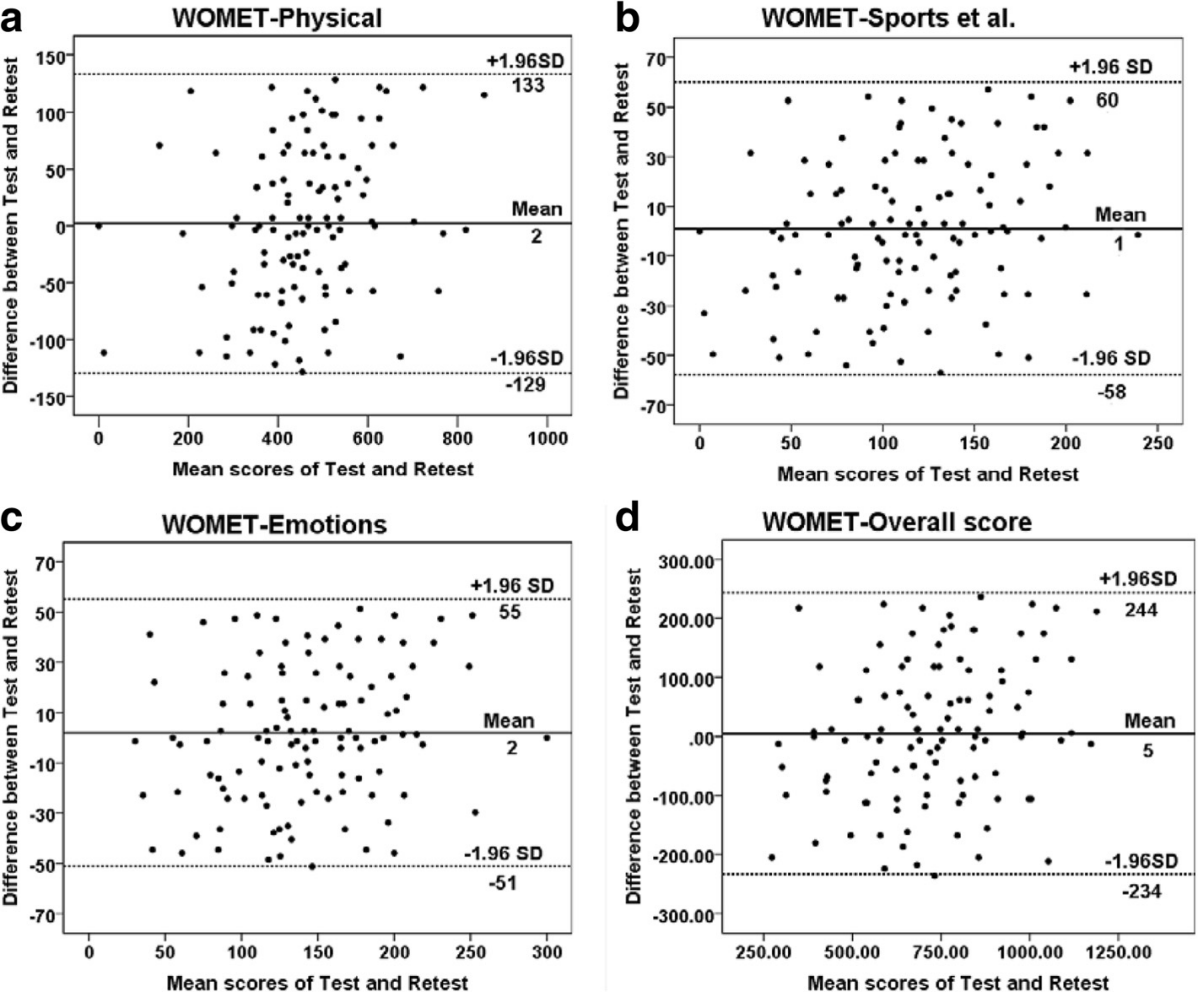
Bland-Altman plots of test-retest reliability of the Chinese version WOMET. The plots are for the **a** *physical*, **b** *sports et al*.: sports/reaction/work/lifestyle, **c** *emotions*, and **d** *overall score* of the Chinese version WOMET. Each *point* indicates the difference in score of Chinese version WOMET for each patient between the two assessments (test and retest). The *dashed line* shows the 95 % (1.96 SD) limits of agreement

The results of reliability are listed in Table 3; the mean subscale scores of test and retest, ICCs, and CIs are all included in it. The ICC for the test-retest was 0.937 (95 % confidence interval, 0.909–0.957) which indicated excellent test-retest reliability. The internal consistency was good for the Cronbach’s *α* which was 0.90. According to Bland-Altman plots, more than 95 % of the points were within the scope of limits of agreement, and the limits of agreement ranged from −234 to 244. The Bland-Altman plots showed no systematic bias and also indicated good reproducibility of the Chinese version WOMET (Fig. 1).

**Table 3:** Test-retest reliability and responsiveness of the Chinese version WOMET

Scale	1st test (mean ± SD)	2nd test (mean ± SD)	3rd test (mean ± SD)	ICC (CI range)	ES	SRM
Overall scale	716 ± 201	716 ± 201	864 ± 217	0.935 (0.909–0.954)	0.86	1.11
Physical symptoms	454 ± 133	457 ± 150	558 ± 145	0.855 (0.844–0.921)	1.06	0.96
Sports/reaction/work/lifestyle	117 ± 49	118 ± 57	157 ± 65	0.846 (0.786–0.891)	1.02	0.88
Emotions	145 ± 48	147 ± 56	149 ± 70	0.867 (0.813–0.905)	0.16	0.10

The 1st test was conducted at the beginning of this research (121 patients), the 2nd test was conducted 2 weeks later to calculate the test-retest reliability (ICC) of the Chinese version WOMET (121 patients), and the 3rd test was conducted 6 months later to calculate the responsiveness (ES, SRM) of the Chinese version WOMET (112 patients)

*ICC* intra-class correlation coefficient, *ES* effect size, *SRM* standardized response mean, *CI* 95 % confidence interval, *WOMET* Western Ontario Meniscal Evaluation Tool

### Validity

According to Pearson’s correlation coefficient analyses, the Chinese version WOMET demonstrated good correlation with physical function (*r* = 0.681) and bodily pain (*r* = 0.636) domains of the SF-36 (Table 4). The WOMET also correlated well with IKDC and the similar domain of WOMAC. The role-emotional (*r* = 0.308) and mental health (*r* = 0.352) domains of SF-36 showed a fair correlation with WOMET. The weakest correlation (*r* = 0.261) was between the stiffness domain of WOMAC and WOMET.

**Table 4:** Construct validity of the Chinese version WOMET

Correlation coefficient *r* or *r*_*s*_ (*P* value)^a^	WOMET subscales			
	Physical symptoms	Sports/reaction/work/lifestyle	Emotions	Overall score
SF-36 subscales				
Physical function	0.684 (<0.001)	0.696 (<0.001)	0.393 (<0.001)	0.681 (<0.001)
Role-physical	0.533 (<0.001)	0.523 (<0.001)	0.213 (<0.021)	0.522 (<0.001)
Bodily pain	0.642 (<0.001)	0.646 (<0.001)	0.345 (<0.001)	0.636 (<0.001)
General health	0.374 (<0.001)	0.395 (<0.001)	0.095 (0.308)	0.374 (<0.001)
Vitality	0.247 (0.007)	0.254 (0.005)	0.571 (<0.001)	0.273 (0.003)
Social function	0.554 (<0.001)	0.539 (<0.001)	0.569 (<0.001)	0.561 (<0.001)
Role-emotional	0.298 (0.001)	0.297 (0.001)	0.602 (<0.001)	0.308 (0.001)
Mental health	0.332 (<0.001)	0.332 (<0.001)	0.644 (<0.001)	0.352 (<0.001)
WOMAC				
Pain	−0.646 (<0.001)	−0.685 (<0.001)	−0.182 (0.049)	−0.639 (<0.001)
Stiffness	−0.274 (0.003)	−0.293 (0.001)	0.037 (0.689)	−0.261 (0.004)
Physical function	−0.715 (<0.001)	−0.734 (<0.001)	−0.118 (0.204)	−0.681 (<0.001)
IKDC	0.746 (<0.001)	0.721 (<0.001)	0.067 (0.468)	0.687 (<0.001)

*WOMET* Western Ontario Meniscal Evaluation Tool, *IKDC* International Knee Documentation Committee subjective knee form, *SF-36* Short Form-36, *WOMAC* Western Ontario and McMaster Universities Osteoarthritis Index

^a^Calculated by Pearson’s correlation coefficient (*r*) or Spearman’s correlation coefficient (*r*_*s*_) of the WOMET with IKDC, WOMAC, and SF-36

### Responsiveness

One hundred twelve from the total 121 patients completed the WOMET questionnaire for the third time. According to Table 3, the scores of all the WOMET subscales improved after surgery. We found that physical symptoms (ES = 1.06, SRM = 0.96) and sports/reaction/work/lifestyle (ES = 1.02, SRM = 0.88) subscales had high responsiveness in patients receiving surgery. The Chinese version of the WOMET showed a good response to treatment.

## Discussion

In this study, the WOMET was cross-culturally adapted into the Chinese version and then showed acceptable psychometric properties (test-retest reliability, internal consistency, construct and content validity, responsiveness) in Chinese people with meniscus pathology.

The test-retest reliability assessed by ICC was excellent for overall WOMET score and all the three subscales (Table 3). This demonstrated that two-time assessments of patient over time remain consistent when there are no changes taken in patient’s health status. According to literature, the value of Cronbach’s *α* higher than 0.7 is acceptable for satisfactory internal consistency. The Cronbach’s *α* coefficient of the WOMET is higher than this threshold with a value of 0.90. No systematic bias together with excellent test-retest reliability indicated a good reproducibility. The result was consistent with previous studies [10, 11, 15].

Construct validity was demonstrated by calculating the correlation among the WOMET, IKDC, SF-36, and WOMAC. There was no gold standard questionnaire that existed. The SF-36 was a general health status-measuring questionnaire, and the WOMAC got similar questions. The IKDC has been validated for meniscus injuries of the knee [24]. Pearson’s correlation coefficient between the overall WOMET and IKDC was 0.687, which indicated that the two instruments measured similar aspects. Lower level correlation (0.067) was observed between the emotion subscale of WOMET and IKDC, since the IKDC does not have the similar item to measure patients’ emotion. As the SF-36 was a more comprehensive instrument than the disease-specific one, we found that the overall WOMET has a higher correlation with physical functioning and bodily pain than with general health, vitality, role-emotional, and mental health subscales of the SF-36 (Table 4). No floor or ceiling effects have been observed, and this indicated a good content validity. All these results are in accordance with other validation studies [10, 11, 15, 23].

Responsiveness refers to the sensitivity of a tool to reflect the changes in the patient’s status after intervention. In this study, we observed significant changes in the overall WOMET score and showed a large effect size (0.86) and standardized response mean (1.11). These values concur well with the findings of earlier validation studies [10, 11]. Based on the results, we found that the Chinese version WOMET was able to detect changes of physical symptoms and sports/reaction/work/lifestyle subscale after surgery with excellent responsiveness.

Several limitations of this study exist. First, the overall amount of the population engaged in this study was not big enough to represent the whole Chinese population. Second, the WOMET questionnaire has been tested only in English and Turkish; thus, psychometric properties in other countries and cultures are unknown. What is more, we only tested patients who underwent arthroscopic surgery, and patients receiving conservative treatments should also be included.

## Conclusions

In conclusion, our results confirmed that the Chinese version of the WOMET questionnaire has good acceptability, reliability, validity, and responsiveness. These findings indicate that the Chinese version WOMET is a valid tool in evaluating health-related quality of life (HRQOL) of patients with meniscal pathology.

## Abbreviations

WOMET: Western Ontario Meniscal Evaluation Tool
HRQOL: Health-related quality of life
OA: Osteoarthritis
ACL: Anterior cruciate ligament
SF-36: Short Form-36
WOMAC: Western Ontario and McMaster Universities Osteoarthritis Index
IKDC: International Knee Documentation Committee score
ICC: Intra-class correlation coefficient
SRM: Standardized response mean
ES: Effect size
